# Twenty Years of High-Resolution Sea Surface Temperature Imagery around Australia: Inter-Annual and Annual Variability

**DOI:** 10.1371/journal.pone.0100762

**Published:** 2014-07-02

**Authors:** Scott D. Foster, David A. Griffin, Piers K. Dunstan

**Affiliations:** 1 CSIRO's Wealth from Oceans Flagship, Hobart, Tasmania, Australia; 2 CSIRO's Marine and Atmospheric Research, Hobart, Tasmania, Australia; University of Vigo, Spain

## Abstract

The physical climate defines a significant portion of the habitats in which biological communities and species reside. It is important to quantify these environmental conditions, and how they have changed, as this will inform future efforts to study many natural systems. In this article, we present the results of a statistical summary of the variability in sea surface temperature (SST) time-series data for the waters surrounding Australia, from 1993 to 2013. We partition variation in the SST series into annual trends, inter-annual trends, and a number of components of random variation. We utilise satellite data and validate the statistical summary from these data to summaries of data from long-term monitoring stations and from the global drifter program. The spatially dense results, available as maps from the Australian Oceanographic Data Network's data portal (http://www.cmar.csiro.au/geonetwork/srv/en/metadata.show?id=51805), show clear trends that associate with oceanographic features. Noteworthy oceanographic features include: average warming was greatest off southern West Australia and off eastern Tasmania, where the warming was around 0.6°C per decade for a twenty year study period, and insubstantial warming in areas dominated by the East Australian Current, but this area did exhibit high levels of inter-annual variability (long-term trend increases and decreases but does not increase on average). The results of the analyses can be directly incorporated into (biogeographic) models that explain variation in biological data where both biological and environmental data are on a fine scale.

## Introduction

A ubiquitous driver of ecosystem function is temperature. The way temperature varies through days, seasons and longer time scales is often a delineating feature of ecological habitats. There has been much recent research in the past two or three decades focussing on just one aspect of temperature, namely long-term temperature rise through long-term climate change [1]–[4]. While long-term climate change is undoubtedly important, it is not the only temperature-related ecosystem driver. Other sources of temperature variability are likely to affect communities and species (e.g. the size of annual temperature cycle).

Variation in temperature is likely to cause wide-spread perturbations to all levels of ecosystems [2], [4]. In the marine realm, a warmer climate will affect the ecosystem by increased water temperatures, changed circulation patterns, a changed oxygen content and acidification [3], [4]. The water's temperature is largely controlled by the boundary between the oceans and the atmosphere (the sea surface) as this is where the radiation balance is controlled. There are, of course, other drivers like advection, river run-off and ice melt but these are of secondary importance and will be local pressures. This argument implies that the sea surface is an obvious quantity to investigate for variation in temperature, including long-term climate change induced responses.

The magnitude of variation in temperature will not be uniform throughout the ocean, including long-term climate change [3], [5]. An understanding of the patterns of temperature variation will aid identification of areas that experience unique environmental conditions. It will also aid identification of those areas that have experienced substantial long-term change. The ability to quantify these variances and changes in the physical properties of the ocean would also be useful to understand the stresses that communities, ecosystems and species endure, and it will give some idea of the change in stresses in the coming decades. The potential change is of direct importance to environmental agencies when they consider various management strategies. Quantification of the sources of variation in temperature could also be a key component in describing species distributions into the future [2], [6].

Using statistical approaches to produce summaries of change for historical data has substantial appeal [5], [7]–[13]. A statistical summary provides a quantification of historical change in climate, with relevant measures of uncertainty. This approach allows identification of areas that have already experienced some change and it defines a delineator for the current patterns of biodiversity or its historical change [13]. It is only available in situations where there is a substantial monitoring effort, through time and preferably through space. In contrast, predictions of physical ocean properties have been made from physical models, at various temporal and spatial scales [14], but are typically limited to large spatial scales which restricts their utility for relating to finer-scale biological data. Physical model predictions, which obey physical properties, can be used for short and long-term prediction. Currently however, quantifying the uncertainty around these prediction is a relatively new field [15], which makes interpretation of predictions from physical models more ambiguous.

We analyse sea surface temperature (SST) for the oceans surrounding Australia. SST is likely to be a very important physical property that has been remotely sensed from satellites for the last two decades. We analyse these data using a flexible non-linear statistical model to investigate the patterns in SST through time. We partition the SST time series into a number of different components that could play important roles in the dynamics of communities and ecosystems. This study differs from previous approaches in its finer spatial scale, the statistical approaches applied and the statistical summaries of trend and variance in climate, all of which are utilised to quantify ecologically relevant aspects of SST variation.

## Materials and Methods

### Data

#### Sea Surface Temperature Data

We use a 20-year long archive of Sea Surface Temperature (SST) imagery produced by the CSIRO Remote Sensing Group using the Advanced Very High Resolution Radiometer (AVHRR) High Resolution Picture Transmission (HRPT) data broadcast by the National Oceanic and Atmospheric Administration (NOAA) Environmental satellites (NOAA9 to NOAA19). The temporal extent of the data used in this study was from the 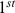 of October 1993 to the 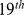 of February 2013. These data are downlinked directly by a number of Australian ground stations and joined together to form long swaths (approx. 55°S to 5°N, 80°E to 190°E) that produce a unique data set for the Australasian region that is both high resolution (approx. 1 km pixels at nadir) and covers a large geographical area. Details on the processing of the downlinked data are given in [16]. For this study we use the 1-day composite images that consist of a grid of cells with 0.042°
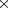
0.036° resolution, which is about 4 km
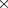
4 km at mid-latitude. With the HRPT data, there are up to 8 views of a point on the surface per day, so the number of raw SST data contributing to each grid-cell is up to 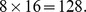


Cloud-clearing [17] was performed but some clouds will remain undetected, especially at night when the visible channel provides no information for discrimination. Any remaining observations contaminated by clouds will result in erroneously low raw-SST data. Conversely, the raw SST data from satellite over-passes between 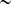
1100 h to 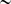
1700 h local time, are sometimes upwardly biased when the winds are particularly weak. This is due to the formation of a thin hot surface layer which can be several degrees warmer than the underlying water. Historically, these sources of excess variation have been mitigated when compiling the 1-day composite images by using the 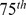 percentile of the raw SST data for each 4 km
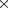
4 km pixel. This is the percentile with the least error against drifting buoy values (D. Griffin pers. comm). We use this percentile approach for the SST data in our study but note that refinements could be made. *Any* method to identify and remove clouds, including the percentile approach, is unlikely to be 100% successful. In particular, they are unlikely to be successful when the number of data per grid-cell is small or when the mechanism for bias (e.g. cloud cover) persists for an entire day. In short, the satellite-derived SST data may contain excess variation and possibly bias. Consequently, analyses based on SST data need to be checked against other sources of data. However, we do not aspire to ‘correct’ the SST data or the signal that it contains to match the other sources of data. For the methods used in this article, correction would have to be performed at the level of each grid cell unless we assume that the bias is similar throughout the entire study area, which seems not to be the case. For most grid cells there is simply not enough alternative data to perform this correction with confidence, so we leave the satellite estimates uncorrected.

#### Long-term Hydrographic Station Data

**Figure 1:**
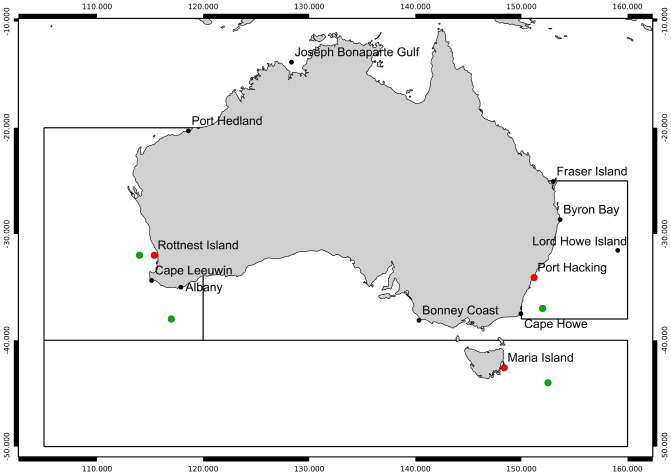
Location of long-term hydrographic stations and example locations. Red dots are hydrographic stations, green dots are example locations and the large boxes identify areas used to compare with drifter data.

The data from four long-term hydographic stations are used for this study. The stations are Maria Island (off the east coast of Tasmania, 42.6°S 148.2°E; Figure 1), Rottnest Island (off the coast of Western Australia, 32.0°S 115.4°E; Figure 1) and two near Port Hacking (off the coast at Sydney, 34.1°S 151.2°E; Figure 1). The Port Hacking stations are located at depths of 50 m and 100 m. All this data is available on the IMOS Ocean Portal (http://www.cmar.csiro.au/geonetwork/srv/en/metadata.show?id=51805).

The Maria Island station has been measured regularly from 1944 until present (October 2012 was the last measurement used here) [8], [18]. The sampling frequency varied: the mean sampling interval is approximately one month and the maximum is just under a year. The Rottnest Island data [19] commences in 1951 and has larger gaps in the record. The largest of these gaps is from 1956 to 1969, but there are also sizeable gaps from 2003 to 2009 and 1997–1999. The sampling interval varied and the mean was approximately 3 weeks (ignoring the large gaps for non-monitoring periods). The Port Hacking 100 m data [18] commences in 1953 and has only one sizeable gap (July 2007 to February 2009). The Port Hacking 50 m data started prior to 1944. The sampling frequency for the Port Hacking data was weekly for the first part of the series (1953–1987) and then approximately monthly (with substantial variation). The Port Hacking data that we obtained had no data later than April 2010.

These data provide a rare opportunity to tie recent trends to those in earlier history. They are not without fault though: measuring instruments have changed through time, as have sampling protocols and personnel. Hence, there may be some small scope for confounding between sampling bias and trend in the time-series. Nevertheless, they do provide an excellent opportunity to validate recent analyses and to extend inferences, qualitatively, through history.

A further complication for comparing satellite derived SST and the temperatures obtained from *in situ* measurements, such as these series, is that the measurements are of slightly different quantities. The satellites measure only the topmost layer of the ocean, less than a millimetre deep, whilst the *in situ* measurements integrate over a metre (at least) of depth. While general agreement is expected, exact agreement is not. In spite of this disparity, we also use observations from deeper time-series from the *in situ* measurements to verify the shallow readings. There should be relatively little change in the series between the depths considered, except for a cooling with depth.

#### Drifter Data

The data from the Global Drifter Program (GDP) (http://www.aoml.noaa.gov/phod/dac/dacdata.php) are also used in this study in a similar manner to the hydrographic stations' data, to check the robustness of our analyses of the SST data. The drifter data, like the hydrographic stations' data, do not measure the same quantity as the satellites and so some discrepancy between the drifter and the SST data is inevitable. The key difference between the drifter data and the other two data sources is that the drifters are mobile and therefore cannot provide a time-series of temperature for a single location. To aid comparison, we define three regions with substantial area and use the data within each region to form a composite (regional) time series. Note that the variation in location, within a region, is not yet accounted for. The regions are: East (bounded by 38°S, 25°S, 150°E and 160°E), South (bounded by 50°S, 40°S, 105°E and 160°E) and West (bounded by 40°S, 20°S, 105°E and 120°E). We did not specify a northern region due to a lack of drifter data. These data can not be compared to the SST data using the same methods as the long-term hydrographic stations due to the mobility of the sensors.

### Data Handling and Statistical Models

The primary goal of the analysis is to produce a map of summaries of the observed SST change in the Australasian region. The SST data set is spatial and temporal and can be thought of a large set of time series, one for each spatial grid cell. Each time series spans a period of approximately 20 years. The SST spatial resolution is high, so there is no need to do spatial interpolation; analyses on individual spatial locations is sufficient. There are almost 2 million non-empty grid cells with more than 750 observation days throughout the entire period which are analysed separately. Each of these 2 million grid cells has a model fitted to it, which is a substantial computational challenge. Any grid cell with less than 750 observation days is not analysed as the amount of information *may* not be sufficient to support the model. This is a conservative approach but it excludes only a tiny proportion of grid cells. Summaries of the individual analyses can be represented spatially to give an idea about spatial variation but neighbouring locations are not incorporated into each grid cell's analysis.

#### Statistical Models and Methods

**Figure 2:**
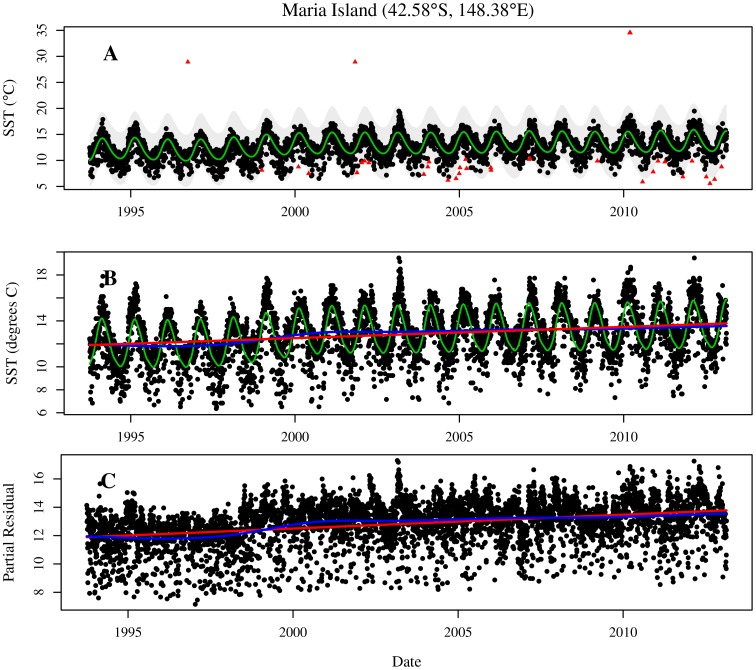
Temporal data for a location near Maria Island, off the east coast of Tasmania (42.57S, 148.36E). Panel A: All data including possible outliers (red triangles). Green line is the robust fit for data validation. Shaded region is the interval that contains data that is likely to be valid. Panel B: Fitted model and model summaries for cleaned data. The lines are: green – fitted values; blue – fitted values excluding seasonal component, and; red – linear summary of long-term trend. Panel C: Cleaned temperature data with seasonal cycle subtracted. The lines are as in panel B.

It is clear from time-series plots of the data for each location (see Figure 2 for an example location) that there is a large amount of temporal variance in the data. Some of this variance is signal, attributable to estimable proceeses, and some of it is noise. The noise appears to be manifested in two components: randomness common to all data, giving random scatter, and; ‘outlying’ data that is due to artefacts, either physical or instrumental. In principle, a statistical model may be able to investigate signal *and* both types of noise. However, pragmatically it may be better to remove any outliers prior to analysis – a statistical model that allows for them would have to make some, possibly severe, assumptions about the nature of the outliers.

We start by describing the statistical model used to describe the signal in the temporal data. It follows the model described in [20] (Section 6.7) and is similar to [10], [21], [22]. We will assume that outliers have been removed as this simplifies notation. The basic principle is that the temperature time-series, for any spatial location, can be decomposed into:

#### Inter-annual variability

This includes the long-term trend and any variability with multi-year time-scale. This is modelled as a smooth function of time 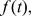 say. Here 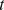 reflects the day since the start of the observation and 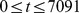 days, where 7091 is the number of observation days included in the study.

#### Annual cycle

This is a periodic function with the same timing and amplitude every year. It is assumed to be a smooth function of day-within-year but not necessarily trigonometric or a function of trigonometric functions. Denote this function as 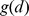 say, where 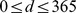 days (or 366 days in a leap year).

#### Residual

All random (and some non-random) deviations from the model's expectation. It includes: a) patterns that occur on a time scale that is shorter than the 1-day data (diurnal effects – a cell is not measured at the same time each day), and 2) non-smooth trends and other model misfit issues. The latter can occur when one of the modelling assumptions fails. An example is when the annual cycle changes abruptly between years, as can happen in an El Niño year, for example.

The components of variation in the time-series data can be formally included into a statistical model, viz
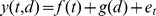
where 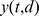 is the SST observation on the 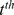 day after the time-series starts (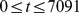 days) that is observed on the 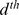 day of the year (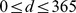). The functional form of the longer-term trend, 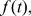 and the seasonal cycle, 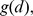 could take many forms. Here, a penalised cubic regression spline is used for 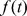 and a penalised cyclic regression spline is used for 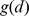 [20]. In both spline terms we use nine knot points. More knots could be used but this increases the computational burden and if increased too far could start detrimentally partitioning variance that belongs at shorter time scales. Other choices of function forms are available for 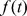 and 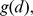 such as linear 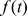 and a low order Fourier approximation for 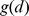 ([21], [23], for example). In a situation like this, where there is no *a priori* assumption of a functional relationship between time and SST, spline formulations offer compelling advantages as they are flexible, smooth but yet allow the incorporation of important aspects of the data (such as periodicity for 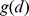). The text books [20], [24] present a detailed argument of when spline methods, and other statistically similar methods, should be preferred.

The model is completed by specifying a probabilistic distribution for the residual effects, 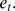 For these data a normal distribution appears to be adequate and was checked at a small number of locations using standard residual diagnostic measures (results not shown). We allow for the possibility of temporal auto-correlation using an AR(1) process, *sensu* [25]. This model is based on the idea that 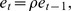 but is parameterised to give 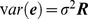 where 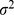 is the variance of the AR(1) process and 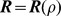 is the usual (scaled) covariance matrix from an AR(1) process. The matrix 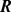 is large and dense, and computation for the model requires that it is repeatedly inverted. To ease this burden, 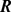 is approximated by assuming that the residuals 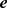 are correlated within a year and independent between years. This gives an approximation of 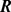 as block diagonal, that is 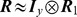 where 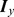 is the identity matrix of size equal to the number of years, 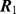 is an AR(1) covariance matrix for the data within the year and 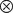 is the usual Kronecker product. The resulting variance matrix is less computationally demanding due to its structured nature and its sparseness. This approximation is taken from [20] (Section 6.7).

The functions 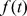 and 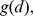 along with the distribution of the residuals 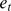 will be governed by parameters that will be estimated from the data. This is performed using restricted maximum likelihood, REML [26], which gives unbiased estimates of the variance parameters.

#### Removing Outliers

The presence of outliers could adversely affect the model's fit and subsequently affect all components of the model's summaries. Their presence will bias the residual variation and possibly (and subsequently) bias all of the model's summaries. We feel that it is better to be slightly aggressive about removal of outliers rather than inflating the risk of inclusion of outliers. Identification of outliers is done in a pre-analysis step. A model with a slightly simplified structure is fitted using robust estimation methods and outliers are defined as those data that are far away from the model's fit [27]. The M-estimation method for statistical models [28] attempts to provide a good estimate of the model's expectation, even in the presence of a moderately large number of outliers [29] (Section 6.5).

The model is similar to that described previously but with a number of simplifications needed to allow robust fitting. First, both the spline functions are regression splines ([29], for example) rather than penalised splines [20]. The number of degrees of freedom for these splines is assumed to be nine, which should provide a smooth model for the amount of data at each location. Second, the residuals are assumed to be independent rather than auto-correlated. Both these assumptions are made to enable standard software to be used [29].

We define an outlier to be any datum that is more than 4 standard deviations away from the robust model's fit. If the data are truly normally distributed (and independent) then it is expected that over 99.9% of the data will lie within this interval. Hence, any observation outside this interval has small chance of being a legitimate observation and a high chance of being an outlier.

This process is demonstrated in Figure 2a. The solid-green line is the robustly fitted model and the grey area is the interval dividing legitimate data (black dots) and outliers (red triangles). In this example, all obviously erroneous data are labelled as outliers.

#### Comparison with *in situ* data

We compare the SST results against the data from the hydrographic stations by comparing the fit of models constructed on each data source. We do not have an SST time-series at the exact location of the station, so we find the nearest four SST grid cells and use the analysis from those for comparison. The exception is the Port Hacking 50 m station that was too close to land to get all four comparative cells. For Port Hacking 50 m we only use the seaward grid cell. We do not expect the stations' data to exhibit large amounts of auto-correlation as the data are less dense through time (almost all are separated by at least two weeks). Hence, for analysis of the historical data, we omit the auto-correlation term. Also, since the series is longer than the SST series we increase the number of knot points for the long-term spline. This was done so that there are (roughly) the same number of knots per year in both data sets. This increase in knots is potentially tempered by the splines penalty, which is the major driver of the spline's flexibility.

The SST data is compared against the drifter data using similar philosophy and methods. However, the analysis needs to be slightly altered to accommodate the fact that the drifters are mobile. First, a comparable SST data is found by finding the nearest SST datum to each drifter datum in time and space. The difference in the two data sets is plotted against time. Also, a generalised additive model, identical to 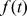 above, is fitted to the difference and is plotted on the difference graph to aid visual interpretation. If there is no difference in the signal from the two sources then this model will be identically zero for all times. The size of departures from zero will suggest some level of bias.

### Model Summaries

The model is interpreted by exploiting the relationship between penalised spline models and mixed models [20], [30]. The inter-annual spline term can be expressed as 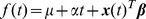 where 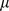 is an intercept, 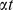 is a linear trend and 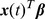 is a spline function whose basis, 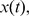 is orthogonal to the linear term. The penalised spline treats 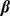 as random and assumes that they are (multivariate-)normally distributed with variance. With this representation a number of useful summaries can be extracted from each location's model. They are:

**ALTT** The average long-term trend. This is the slope of the dotted-red straight line in the Figure 2b. It is measured by 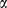 in the mixed model representation.

**ALTT SE** The standard error of the average long-term trend. This gives a measure of uncertainty in the long-term trend estimate and indicates how much it can vary depending on the observed data. It is estimated via the relevant element of the inverse of the Fisher information matrix. It measures only the amount of information in the historical data and does not provide a measure of predictive performance for future events.

**AvSST** The average SST over the time period. This is calculated as the SST on the 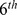 of June 2003, the data's mid-date, using the long-term average trend only. It does not contain any annual component and hence will reflect the average long-term SST. The date of calculation is arbitrary and any day could be used. However, a date at the centre of the data has the advantage of having the smallest level of uncertainty.

**AvSST SE** The standard error of the average SST estimated from the inverse of the Fisher Information matrix.

**Trend RMSE** The amount of non-linearity in the long-term trend. This is measured as the root mean-square error (RMSE) between the fitted long-term spline and the average long-term trend. Formally, it is 
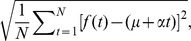
 where 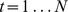 indexes the days in the study. Informally, the RMSE can be thought of as a standard deviation. This summary measures the amount of departure of the blue line from the red line in Figure 2b.

**Annual RMSE** The amount of annual variability. This is measured as the RMSE for the fitted cyclic spline. It measures the amount of departure from the green line to the blue line in Figure 2b.

**Residual SD** The level of unexplained variability in the data. This is measured as the residual standard deviation (SD), 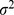 and is obtained directly from the fitted model. It measures the departure from the data to the solid-green fitted line in Figure 2b.

**e-fold time** The short term (temporal) autocorrelation of one datum to its neighbouring days data. In the model this is parameterised as AR(1) parameter, 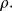 However, we choose to display it as the 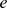-fold time – the lag in days before the temporal correlation is equal to 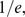 where 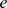 is Euler's number (approximately 2.718). We note that 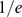 is arbitrary and any choice could be used. However, all choices are (monotonically) related and will produce similar maps that differ only in scale.

These summaries are calculated for each of the grid points with sufficient data. This produces a set of summaries for each of approx. 2 million grid locations. We display each of these summaries graphically as a map. In addition to the maps of estimated ALTT and its standard error, we also present a map of 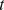-statistics. These statistics are defined as the estimated ALTT/ALTT SE for each spatial location and gives an indication of how far the average long-term trend is from zero.

## Results

The SST data set is 20-years long, which is substantial for a geophysical data set, especially over this geographical range. However, it does not span climatological scales. The long-term trend over the 20-year period is a combination of the trend that can be expected to persist into the future (the *secular* trend) and other (possibly cyclic) trends that could change or reverse over the coming decades. It is the secular trend that is of long-term interest but over the time scales examined here, the decadal variation in SST change is likely to be larger. The SST data set does not have a sufficient time span to completely remove the decadal signals from the secular rate of change. Thus, the trend given by 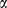 will be the combination of secular and decadal signals.

The fitted models appear to capture the important aspects of the variation in the time series. For example, the long-term trend reflects the inter-annual fluctuations and the annual trend captures the seasonal cycle in the data. The decomposition, illustrated in Figures 2a and 2b, is obviously useful but it is not perfect. The imperfections are attributable to slight variation from modelling assumptions to reality. As an example, consider Figure 2c, which shows the SST data adjusted by subtracting the fitted annual cycle, and the components of the long-term trend. If the fitted annual trend component is adequate then there should be no annual pattern in the adjusted SST data. This is not the case and the slight misfit appears to arise from the progression of the annual pattern to differ between year to year in both timing and in magnitude. We do not believe that this is problematic for the inferences we draw from the data, especially when the entire continent is considered rather than a particular grid cell and could be resolved with more data in the future. The major components of the systematic variation are captured.

### SST average long-term trend (ALTT)

**Figure 3:**
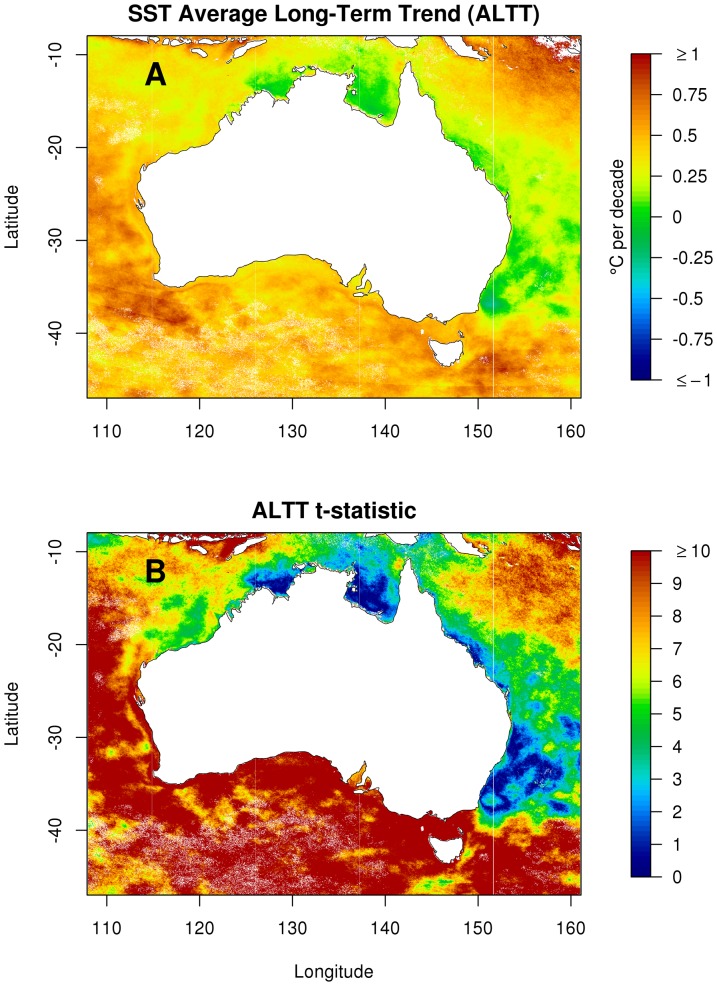
Maps of estimated model summaries for SST change. The map in panel A is for estimated SST average long-term linear change (denoted by 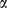 in the text and given acronym ALTT, units are °C per decade). The map in panel B is the 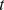-statistic of estimated linear change (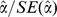) for each point.

We estimate the ALTT of SST in Australian waters to range from about −0.2° per decade to +1° per decade (Figure 3a), in the interval from 1993 to 2013. The estimates near the extremes of this range could be due to data errors or artefacts or the interaction between secular trend and decadal variability. Possible artefacts include the inclusion of pixels nearer to land in the latter part of the time-series, which can artificially inflate a grid-cell's ALTT if it is very close to the coast.

The most extreme result (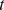-statistic of 7 or more) is that the ALTT for the 1993–2013 period was between 0.4° and 0.6° per decade for much of the area south-west of a line passing through Port Hedland on the north-west coast and Cape Howe at the south-east corner of the Australian mainland (Figure 3a, but see Figure 1 for location names). These values are well in excess of the global averages [7], and are considerably higher than estimates for this region (over longer intervals, see [19]). The two regions within this area of greatest ALTT, relative to their standard error, are near Cape Leeuwin at the southwest corner of the mainland, and east of southern Tasmania, near 43°S 151°E. Both of these regions are affected by warm, southward-flowing boundary currents, the Leeuwin Current on the west and the East Australian Current on the east. Both locations lie within the Subtropical Convergence Zone.

An interesting detail of Figure 3 is that the edge of the continental shelf is clearly recognisable. In the time-period of the data, ALTT was greater seaward of the 200 m isobath than landward of it. This suggests that at least some of the change can be attributed to increased advection of warm tropical waters, which flow south to Cape Leeuwin and then east, almost exclusively seaward of the 200 m isobath [31]. These areas also show up in the other model summaries, to lesser and larger extents.

**Figure 4:**
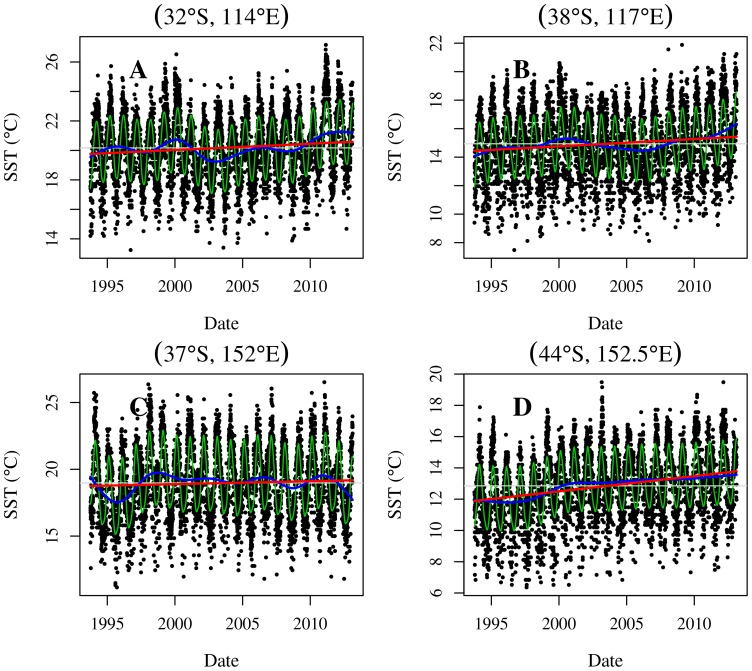
Examples of time-series data and model fit at four isolated locations. Black dots are SST observations, green line is the fitted model, blue line is the fitted long-term trend, red line is the average long-term trend and grey line is the overall mean of the data.

The time-series of SST at 4 key locations, specified in Figure 1, are given in Figure 4. These illustrate important features of temporal summaries in Figure 3. Two of the example locations are within the south-west region of high ALTT: one ∼150 km west of Perth and one 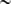
150 km south of Albany (Figures 4a and 4b but see Figure 1 for locations). At both locations the inter-annual variability is certainly not monotonic, but features a downward trend after the minor peak in 2000, followed by a fairly steady rise from about 2004 off Perth and 2006 off Albany. SST reached unprecedented heights in early 2011 off Perth [32] and this is evident in our results, but modelled trend has remained high through to the end of the series. This is in spite of the reduction off Perth of the summer values in 2012 and 2013 compared to 2011 (Figure 4).

The east coast of Australia shows similar patterns. The area east of Cape Howe shows a large spike in SST in 2011, but on average only a very small increase in SST over the 20 years sampled (Figure 4c). In contrast, the high ALTT in the water east of Tasmania does reflect a fairly monotonic increase of the inter-annual trend (Figure 4d). Both these inter-annual trends rise most quickly from 1997 to 2002 and feature a slight cooling trend at the beginning of the series. The difference between the two example locations can be seen in the ALTT (Figure 3a); much of the East coast of Australia shows a marginal warming trend (with low values for the t-statistic), whereas the area to the east of Tasmania shows pronounced warming over the same time period.

The least smallest ALTT, with respect to variability (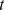-statistics less than 3), are along the northern and eastern seas between Joseph Bonaparte Gulf (13°S 127°E) and Cape Howe. The Tasman Sea off the south east coast of Australia (latitudes 28°S to 37°S) is the largest region of low 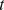-statistics. In both these areas we have the least certainty as to whether SST rose and fell. An example time series for this area is given in Figure 4c, which features an extended cold period in 1995–1996, a subsequent rapid rise followed by relatively little change until 2012 and 2013 which are cooler than immediately earlier years.

We estimate that the surface waters of the Great Barrier Reef warmed at an ALTT of between 0.1 (southern and central regions) and 0.3° per decade (northern and far northern regions). This is somewhat more than the 0.08° per decade area-average previously reported [33] for the 1985–2009 period. Notably, the waters just offshore from the continental shelf had a greater ALTT than the waters inside the 200 m isobath, for most of the length of the isobath.

### Average SST (AvSST)

**Figure 5:**
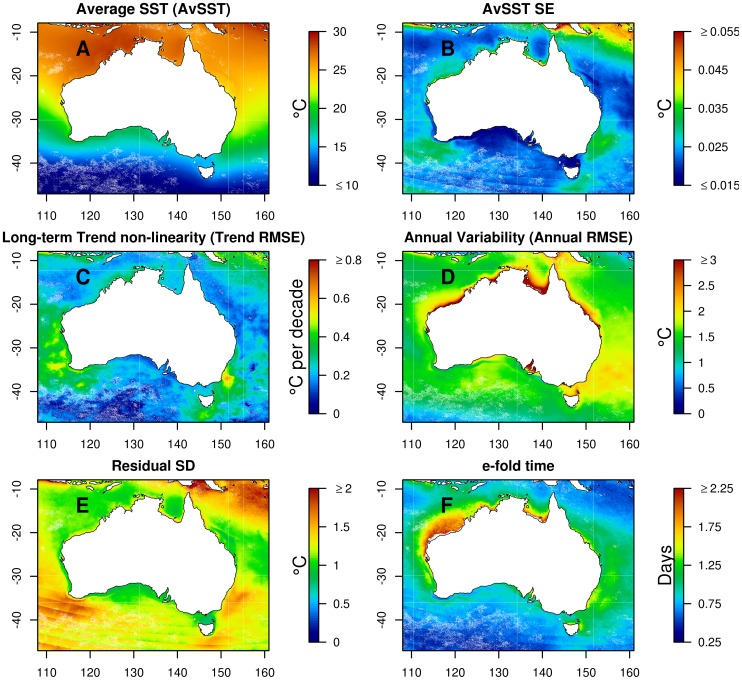
Maps of summaries from estimated models. The maps are for: panel A – average SST (AvSTST) for the study period; panel B – standard error for the average SST (AvSST SE); panel C – variability in long-term trend (trend RMSE); panel D – variability in annual trend (annual RMSE); panel E – residual standard deviation, and; panel F – temporal (day-to-day) autocorrelation (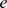-fold time).

There is a clear, and unsurprising, north-south decline in AvSST throughout the region (Figure 5a). There are some departures from this general pattern however. The area affected by the Leeuwin Current, which brings warmer water from the north of the western boundary, is warmer than the surrounding water. The narrow band, which is bounded by the east coast of Australia near Sydney and the oceanic area affected by the East Australian Current (EAC), is cooler than the seaward waters. This is due to the EAC not affecting the inshore waters as regularly as the seaward waters. The lagoon of the southern Great Barrier Reef off the coast of Queensland is cooler, on average, than the oceanic waters seaward.

### Inter-annual variability of SST (Trend RMSE)

Many of the patterns shown in the ALTT (Figure 3a) can be explained by examining the patterns of trend RMSE (see Figure 5), which describes the amount of non-linearity in the inter-annual trend. Trend RMSE is high for substantial sections of the south west and south east coasts (approx. 0.4°C and 0.5°C respectively). These are the regions also associated with substantial warming (Figure 3) and are under the influence of the Leeuwin Current and the East Australian Current respectively. In both these areas warm-core eddies are prevalent. Trend RMSE is just 0.1°C to 0.2°C for northern Australia and the Great Australia Bight – regions not influenced by the continent's largest seasonal currents (Leeuwin Current and the East Australian Current). In addition the following observations are noteworthy (see Figure 1 for geographical locations):

- Trend RMSE along the path of the EAC from Fraser Island (25°S) to Byron Bay (29°S) has a much lower value than south of Byron Bay. This indicates that the extra variability south of Byron Bay does not result from a purely advective process (as might be reasonably hypothesised to be the case over short distances).
- SST along the south eastern coastal margin, inshore of the EAC, has fairly low trend RMSE as far south as 34°S. At this point it suddenly increases to 0.5°C. Indeed, the coastal stretch with the greatest trend RMSE in Australia is between Sydney and Woolongong (34°S to 34.5°S). Here the EAC frequently comes very close to the coast (as it did on the 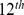 of October 2013: http://oceancurrent.imos.org.au/SNSW/2013101206.html).
- Trend RMSE has a local minimum near Lord Howe Island, presumably because the Lord Howe Ridge inhibits the passage of warm-core and cold-core eddies.
- The Tasmanian continental shelf has much lower (0.2°C) trend RMSE than the adjacent continental slope (0.4°C). There is a sharp boundary along the 200 m isobath, indicating that the Tasman leakage [34] is strongly constrained to follow the continental slope.
- There is relatively little (approx. 0.2°C) trend RMSE between north-west Tasmania and the western Great Australian Bight (125°E), even though the Leeuwin Current extension, the South Coast Current, Bonnie Coast upwelling zone and the Zeehan Current are phenomena that respond to inter-annual forcing. However, these phenomena are visible in the other summary statistics.
- Across the tropics, the area with the greatest trend RMSE is the Joseph Bonaparte Gulf and the adjacent shelf. This is also the region of minimum average long-term trend, as mentioned previously.

### Annual variability of SST (Annual RMSE)

The amount of annual variation (measured by the annual RMSE for the fitted cyclic spline) varies substantially over the region (Figure 5d). Some areas exhibit relatively low amounts of annual RMSE (approx. 0.75°C). The oceanic environment in the extreme south-west of the study region (south of 42°S) shows very little seasonal variation. The Bonney coast (38°S, 138E) is of particular interest. It is an area associated with high productivity for marine fauna. It has very low values of seasonal variation because is it strongly influenced by the Leeuwin Current (a warm winter current) and the Bonney upwelling (a summer upwelling). These two phenomena reduce the difference between winter and summer SST in this region. There are no other areas on the coast of Australia with such low seasonal variability.

Some areas exhibit much larger annual RMSE (approx. 2.25°C and more), in particular sheltered areas along the coastline, such as bays and gulfs throughout the north and in South Australia, and the Great Barrier Reef Lagoon. These areas have high annual RMSE due to the combined effect of solar radiation, shallow water and low circulation. The Tasman sea shows about twice the level of seasonal RMSE of the other open-ocean regions (Tasman sea annual RMSE ranges from 1.75°C to 2.25°C).

### Unexplained variation in SST (residual SD)

Variation in the amount of residual standard deviation (SD) is influenced by two underlying processes: the true day-to-day variability in the SST at any location, and measurement error (due to atmospheric conditions and data processing). The map of residual SD is given in Figure 5e. The map shows an area of very high residual SD (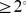
C) near Papua New Guinea and extending towards Vanuatu. This is an area that is far from the receiving stations and is an area of high cloud prevalence, especially during the summer monsoon season. Both these facts imply that the measurement error is likely to be high in this area. The large areas of water off the east coast (around 35°S, 152°E), the south-west coast (around 37°S, 115°E) and off southern Tasmania (43°S, 148°E) with a substantial residual SD, around 2°C. These are all areas under the influence of strong seasonal currents and part of the high residual SD may be attributable to the model not picking up variation in the timing of the seasonal currents and their strength. The striations in residual SD, most prominent in the southern ocean, are due to the methods for pre-processing of the raw data to remove cloud.

### 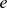-fold time (temporal autocorrelation)

The temporal autocorrelation (day-to-day) in Figure 5f, quantified as 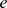-fold time, is greatest on the continental shelf off the north west coast (around 19°S 117°E) and near the coast in the Gulf of Carpentaria. In both cases the e-fold time is around 2.2 days. The areas off south eastern Australia and eastern Tasmania have elevated, but not extreme, autocorrelation (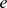-fold time of around 1.25 days).

### Comparison with hydrographic stations

**Figure 6:**
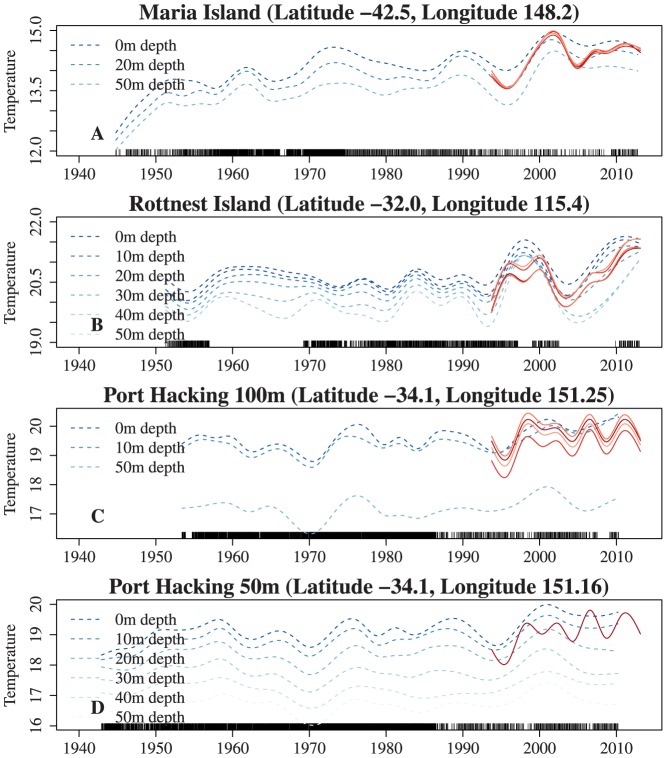
Modelled long-term trends from the four long-term stations. The dashed blue lines are the trends for different depths. The solid red lines are the trends from the four nearest grid cells from satellite SST. The tick-marks on the x-axis show when each observation was taken.

The historical data and the associated SST analysis show similar patterns (Figure 6). The Maria Island series shows a noticeable increase in temperatures throughout the period but are accentuated by two periods of more rapid change. These are (approximately) from 1944 to 1950, and from 1995 to 2002 (Figure 6a). The latter period is matched by the satellite data, as is the flattening off after the increase. At other times there is little to no temperature increase.

The Rottnest Island long-term trends show periods of warming and cooling, for which there good agreement for trends from both SST data and historical data. The historical data suggest that the recent changes in temperature are larger than anything that has happened through measured history. However, we feel that this is not definitive due to the gaps in the time-series.

The Port Hacking long-term trends (Figures 6c and 6d) indicate that there have been a number of periods of warmer temperatures and periods of cooler temperatures. The SST data near the Port Hacking stations also has peaks and troughs of SST, however they occur on a much shorter time scale. Both data sources indicate that the series show an increase in SST from about 1995 but the length of increase differs and the SST model has additional peaks and troughs not shared by the stations' data.

### Comparison with drifter data

**Figure 7:**
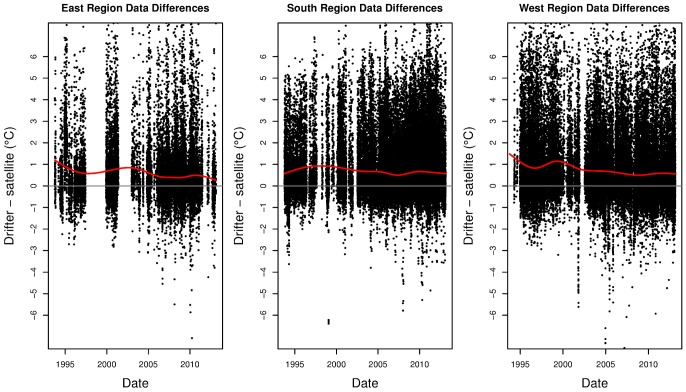
Difference in temperature from drifters and satellite-derived SST. Points are drifter temperature minus the nearest (spatially and temporally) SST observation. Red line gives the penalised spline fit for the points and grey line is for 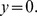 If the red line coincided with the grey line then the two data sources would have the same expectation.

The difference between the drifter data and the matching SST data is given in Figure 7. Also given is the mean prediction from the fitted model. The time-varying mean difference is always above zero, which indicates that the satellite consistently measures cooler temperatures than the drifters. The amount of bias changes over time with the more recent measurements generally being less different than the earlier measurements. The bias appears to change over time and the amount of change depends on the region. Both the east and west regions showed a decrease in bias of around 0.2°C per decade, while the south region's decrease was much more modest (around 0.05°C per decade).

All three regions showed some reduction in long-term bias, indicating that the estimates of average long-term trend from the SST data are likely to be over-estimated when compared to those estimated from drifter data. However, the amount of over-estimation depends on the location of the grid cell. There is no single adjustment that can be made to all grid cells.

## Discussion

There are some marked patterns in the maps of the model summaries (Figures 3 and 5). Our analysis of average long-term trends (ALTT) show many similarities to previous studies using longer term records [8], [19], [35]–[37], but there are some important differences. The most notable ones are the areas of higher ALTT in south-west Australia and also off eastern Tasmania. Previous studies have identified these areas as regions where the climate has changed [8], [19], although both studies reported changes less than found in our analysis. Some previous work has identified similar regions [36], [37] on the west coast of Australia but identified the east coast of the Australian mainland rather than the east coast of Tasmania as a region of rapid change. There are a number of possible reasons for the discrepancies: 1) the time period analysed (the Rottnest SST series has increased substantially in the time period matching the SST data, see Figure 6b); 2) a different SST product was used, which is processed differently; 3) the east Tasmanian study [8] analysed data from a mooring on the continental shelf, an area that did not exhibit the severe warming and so could not detect the higher change (see Figure 3a).

The area to the east of mainland Australia is well studied. It is known to be dominated by the East Australian Current (EAC, see [38] and references therein), which is a strong southward current. It exhibits very low ALTT, meaning very modest long-term changes in average SST (Figure 3a) and the change is not substantial at any location, with respect to the variability in the estimate of change. In addition there is high inter-annual variation (measured by trend RMSE, see Figure 5c). This is an area that has been identified as having rapid change due to long-term climate change, but appears to be more associated with high variability rather than rapid change [39]. This implies that the SST in the EAC governed area might oscillate smoothly, at least within the period investigated, and could be caused by smooth oscillations in the strength of the EAC – when the EAC is strong the SST is warmer and when it is weak then the SST is cooler.

Our analysis shows more than just average long-term trends, and this has implications for future studies using historical data (on any oceanographic system). There is substantial variability in the temporal temperature data and some of this is systematic (e.g. seasonal cycles and inter-annual trends). There is also substantial spatial heterogeneity (both small and large scale) in all the statistical summaries. All these sources of variability are important for the biogeography of the region and having these summaries, at high resolution, should aid future cross-disciplinary research. In this work, the spatial patterns in temperature series are highlighted for the oceans around Australia. We expect that other regions would exhibit similar levels of spatial and temporal variability. We recommend against spatial and temporal aggregation prior to analysis. Such aggregation can confuse fine-scale variation with uncertainty.

A key difference between this study and previous work has been our utilisation of advanced statistical tools that allow for both a linear trend and an understanding of the variance around that trend, both inter annually and annually. The climate impact on ecosystems from rapid, non-secular, trend is likely to be substantially different to gradual long term change and it is important to understand where the different patterns are occurring.

We compared the results from the analysis of the spatially and temporally dense SST data to two alternative sources of data, four hydrographic stations and drifter data in three regions around the continent. The station data and the drifter data are both *in situ* measurements and hence, some minor disagreement between the results between these sources and the SST data should be expected. Broadly speaking, there was good agreement in nearly all the series, with similar patterns exhibited. The notable exceptions were the Port Hacking hydrographic sites. Also, temperatures from the satellite SST data tended to be lower than that from the drifter temperature data, which is slightly accentuated near the start of the time series. This will produce a higher estimated ALTT from the satellite data than from the drifter data. There are several potential reasons for this discrepancy between the two data sources. They include time-varying bias in the instrumentation, time-varying bias in the processing methods, time-varying sampling bias and how these interact with the analysis methods. Biases may occur in both the satellite (AVHRR HRPT) data and the drifter data.

In our statistical approach we attempt to detect and remove individual datum that have an elevated chance of being outliers, considering a single location at a time. This seemed successful in all test locations that we investigated. However, it will only detect problem data within a location – it will not detect problems *between* locations. If problem locations exist, they may cause spatially non-smooth patterns in the summary statistics. This could be evident in the SST results presented here, particularly in striations in the residual variance and the 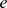-fold time summary statistics in the Southern Ocean. We suspect that these arose from the manner in which the raw data were processed to produce the 1-day composite product. Investigation of this artefact is outside the scope of this article. However, we note that a statistical approach, like that based on a robust estimation method, could be expanded to include multiple locations. It is our firm belief though that this should only be used after an investigation of other possible reasons have been exhausted.

The statistical model utilised in this work is a simple description of the complex patterns of SST through time, one that could be fitted to a large number of time series (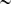
2 million locations). The model has the strength that many facets of the SST time-series can be modelled flexibly, without making too many unnecessary assumptions and accounts for the inherent auto-correlation in the data. The two situations where this is particularly important are if the long-term trend is non-linear, or if the seasonal cycle cannot be described effectively by a first or second order Fourier approximation. These types of model mis-specification have the effect of inflating the residual variance and the model's standard error, giving an overly conservative representation of the uncertainty in the data. Failing to account for temporal autocorrelation will have the opposite effect, it will make measures of uncertainty overly optimistic.

Representing a non-linear trend using a straight line, which is regularly performed [9], [19], [36], may give adequate estimates of the average slope in many cases. However, if the non-linear trend is such that the earliest or latest observations are the most deviant from the linear approximation, then these points will have an unwarranted amount of influence on the model's fit ([40] for example). An alternative to linear or non-linear models is to simply difference the averages for a period late in the series and a period early in the series. At first glance this approach circumvents the modelling choice. However, it *does* assume that there is no trend within those periods. It is also highly dependent on the way in which the period boundaries are chosen.

## Conclusion

We have analysed 20 years of high-resolution Sea Surface Temperature data in order to produce a set of data summaries with unprecedented spatial resolution and statistical rigour for the Australasian region. The summaries include the output from statistical analyses and are available digitally at http://www.cmar.csiro.au/geonetwork/srv/en/metadata.show?id=51805. We have also compared our analyses with available *in-situ* data and found that there was good broad agreement between the two sources. The statistical summary that is possibly of widest interest is our estimate of the average long-term trend in SST over the past 20 years. Our estimates of the average long-term trend in SST do differ from other published estimates. Reconciling those differences, however, is beyond the scope of the present study. We note that simply changing the number of years analysed is enough to change the average long-term trend in most analyses (due to the inter-annual variation). We expect our analysis to be more robust, as this randomness is incorporated in the model, but we doubt that it is infallible as we assume that this randomness is smooth through time. All the statistical summaries presented in this paper have been estimated before but never at this spatial resolution, or so carefully. The summaries are provided to the community for further analysis.
